# Frailty and mobility in hospitalized older adults: an analytical cross sectional study

**DOI:** 10.1590/0034-7167-2024-0570

**Published:** 2025-11-03

**Authors:** Maria Helena Lenardt, Clovis Cechinel, João Alberto Martins Rodrigues, Tissiane Bona Zomer, Maria Angélica Binotto, Larissa Teleginski Wardenski

**Affiliations:** I Universidade Federal do Paraná. Curitiba, Paraná, Brazil; II Fundação Estatal de Atenção à Saúde. Curitiba, Paraná, Brazil

**Keywords:** Early Ambulation, Frailty, Hospitalization, Aged, Mobility Limitation., Ambulación Precoz, Fragilidad, Hospitalización, Anciano, Limitación de la Movilidad.

## Abstract

**Objectives:**

to analyze the association between frailty and mobility among hospitalized older adults in general medical wards.

**Methods:**

this analytical cross-sectional study was conducted in a hospital in southern Brazil. Data collection involved a sociodemographic and clinical questionnaire, physical frailty phenotype markers, and the Perme Intensive Care Unit Mobility Score. The study included patients aged 60 years or older admitted to general medical wards. Exclusion criteria were critical illness, intensive care unit indication, pending transfer, or droplet precautions. Descriptive statistics, Tukey’s test, and the test for equality of proportions were used.

**Results:**

of the 547 participants, 46.07% were prefrail and 36.74% were frail. A significant difference in mobility scores was found between frail (15.2 ± 8.95) and non-frail (27.6 ± 5.05) older adults (p < 0.001), and between frail (15.2 ± 8.95) and prefrail (25.9 ± 6.45) individuals (p < 0.001).

**Conclusions:**

frail older adults hospitalized in general medical wards exhibited significantly lower mobility levels compared to their non frail and prefrail counterparts.

## RESUMEN

**Objetivos:**

analizar la relación entre la condición de fragilidad y la movilidad en personas mayores hospitalizadas en salas generales.

**Métodos:**

estudio transversal analítico realizado en un hospital del sur de Brasil. Se usaron cuestionarios sociodemográfico y clínico, marcadores del fenotipo de fragilidad física y el *Perme Intensive Care Unit Mobility Score*. Se incluyeron personas de 60 años o más hospitalizadas en salas generales, excluyéndose casos críticos, con indicación de cuidados intensivos, en espera de traslado o bajo precauciones por gotas. Se emplearon estadística descriptiva, prueba de Tukey y prueba de hipótesis de igualdad de proporciones.

**Resultados:**

de los 547 participantes, el 46,07 % eran prefrágiles y el 36,74 % frágiles. Hubo asociación significativa entre los puntajes de movilidad de los grupos frágil (15,2 ± 8,95) y no frágil (27,6 ± 5,05), p < 0,001, así como entre frágil (15,2 ± 8,95) y prefrágil (25,9 ± 6,45), p < 0,001.

**Conclusiones:**

las personas mayores frágiles mostraron menor movilidad durante la hospitalización que las no frágiles y prefrágiles.

## INTRODUCTION

During the aging process, muscles, bones, and joints undergo physiological changes that may affect mobility and, consequently, impact the independence of older adults^(1)^. As it encompasses a set of signs and symptoms, mobility in older adults is considered a geriatric syndrome^(2)^. It serves as a marker and predictor of physical ability, independence, morbidity, and mortality in this population^(3)^.

Given its high prevalence as a geriatric syndrome, the diagnosis, assessment, and treatment of mobility limitation are essential. Limitations due to decreased bone and muscle mass^(1)^ have been the subject of several literature reviews aimed at analyzing associations with other variables, such as female sex^(4,5)^, low physical activity^(5,6)^, socioeconomic status, nutritional status^(7)^, and sensory, cognitive, and physical impairments^(4-6)^. However, these reviews have primarily focused on community-dwelling older adults.

In addition, regarding clinical conditions, most published studies assess the relationship between mobility limitation in older adults and multimorbidity^(8,9)^, as well as noncommunicable diseases such as neurological^(10)^, cardiac^(11)^, and respiratory conditions^(12)^, depression^(13)^, diabetes and osteoarthritis^(6)^, stroke^(14)^, and dementia^(4,15,16)^.

The association between mobility and physical frailty during hospitalization in older adults has not been thoroughly explored in national and international studies. Physical frailty is one of the main determinants of functional decline and early mortality in this age group, as it is characterized by a clinical state of increased vulnerability to internal and external stressors^(17)^.

Mobility decline occurs in the context of acute illness and hospitalization and, under such circumstances, may be redefined as being confined to bed or transferring only from bed to chair, resulting in reduced ambulation during the hospital stay^(2)^. Functional decline associated with hospitalization in older patients prolongs hospital stays and increases the post-discharge care burden, the risk of disability and mortality, and medical costs^(18,19)^.

Given that hospitalization is associated with additional decline in functional capacity, the findings of this study may support the development of interventions to mitigate such decline in physically frail older adults.

## OBJECTIVES

To analyze the association between physical frailty and mobility among older adults hospitalized in general medical wards.

## METHODS

The study report followed the STROBE checklist (STrengthening the Reporting of Observational Studies in Epidemiology)^(20)^.

### Study design and setting

This analytical cross-sectional study was conducted among older adults evaluated within the first 48 hours of admission to the general medical wards of a medium-complexity hospital specializing in geriatric care, located in the southern region of Brazil.

### Population, inclusion and exclusion criteria

The study population consisted of older adults hospitalized in general medical wards. Individuals aged 60 years or older who were admitted to medical or surgical wards were included. Those in critical condition-such as patients awaiting admission to the intensive care unit-or those under droplet precautions were excluded.

### Sample size and data collection

To determine the sample size, a prevalence of 50% was used to ensure the largest possible number of participants. The sample size calculation was based on a known population of older adults hospitalized in 2019 (n = 4,146). A 95% confidence level and a 5% sampling error were defined. Based on these parameters, the minimum required sample size was 352 individuals. In this study, a sample of 547 participants was adopted to achieve a sampling error of less than 5%.

Before administering the instruments, members of a research group-including undergraduate students, multidisciplinary residents, and graduate students from a federal university-received theoretical and practical training to standardize data collection and reduce the risk of potential bias. Data collection took place between March 2022 and July 2023 in the hospital’s general medical wards. On average, it took approximately 30 minutes to apply the data collection instruments.

Cognitive screening was first conducted using the Mini-Mental State Examination (MMSE)^(21)^, which is validated for use in Brazilian Portuguese^(22)^, to identify older adults capable of answering the questionnaires and signing the Informed Consent Form (ICF). In cases where the older adult exhibited cognitive impairment and/or communication difficulties, the accompanying caregiver was invited to answer the questionnaires and sign the ICF, while the tests were administered to the older adult. If the accompanying person at the time of data collection did not feel capable of answering the questions, the primary caregiver was contacted to ensure the reliability of the information. In this study, the primary caregiver was defined as the person who provides direct care to the older adult and has lived with them for at least three months.

Data collection began with the administration of a sociodemographic and clinical questionnaire, which included the following variables of interest: sociodemographic variables - age, sex, race/ethnicity, marital status, education level, household composition, and income; and clinical variables - asthma, stroke, cancer, dementia, depression, diabetes mellitus, dyslipidemia, Parkinson’s disease, chronic kidney disease, chronic obstructive pulmonary disease, epilepsy, benign prostatic hyperplasia, hypertension, hypothyroidism, and heart failure.

Physical frailty was classified according to physical frailty phenotype markers: reduced handgrip strength, slow gait speed, unintentional weight loss, low physical activity level, and self reported fatigue or exhaustion. Participants presenting one or two of these components were classified as prefrail, while those with three or more were considered frail^(23)^.

Mobility scores were obtained from the electronic medical records by retrieving physical therapy notes recorded on the same day as the other data were collected. The Perme Intensive Care Unit Mobility Score provides a total score (ranging from 0 to 32) that assesses the patient’s overall mobility status.

The Perme Score evaluates mobility across seven main categories:

- The first refers to mental status, assessing alertness and ability to follow commands.
- The second addresses potential mobility barriers, including the use of invasive or noninvasive mechanical ventilation, the presence of pain, intravenous lines, catheters, a nasogastric tube, and chest drains.
- The third evaluates functional strength, examining the patient’s ability to perform shoulder flexion of at least 45° and hip flexion of at least 20°.
- The fourth covers bed mobility, including moving from the supine to the seated position and maintaining static sitting balance at the bedside, with varying levels of therapist assistance if needed.
- The fifth involves transfer ability, assessing the capacity to stand up from a seated position, maintain static balance, and perform bed-to-chair transfers.
- The sixth category evaluates gait, observing the patient’s ability to walk with or without assistive devices and at different levels of therapist assistance.
- Finally, the seventh category measures endurance, defined as the distance walked over a two-minute period, with or without assistive devices^(24)^.

### Data analysis and processing

Descriptive statistical methods were applied to analyze the sociodemographic characteristics according to frailty status. Measures of central tendency and dispersion were calculated, including means, standard deviations, medians, maximum and minimum values, and frequency tables. The Tukey test was used to compare the mean scores of the Perme Index and its subindices across frailty groups. To compare the proportions of Perme Index scores among the different frailty categories, the test of equality of proportions was used. All hypothesis tests were performed with a significance level of 5%. Statistical analyses were conducted using R software, version 4.2.2.

### Ethical considerations

This study is part of the parent project titled “Physical frailty and clinical, functional, psychosocial, nutritional, and care demand outcomes in hospitalized older adults”. It was approved by the Human Research Ethics Committee of the Health Sciences Sector at the Federal University of Paraná and by the Human Research Ethics Committee of the Curitiba Municipal Health Department. Formal consent was obtained from the older adult and/or their companion using the Informed Consent Form (ICF) prior to study initiation, in accordance with Resolution No. 466/12. Confidentiality and data privacy were ensured in compliance with Resolutions No. 510/2016 and No. 580/2018, as well as other resolutions issued by the Brazilian National Health Council/Ministry of Health, which were in effect at the time of the study.

## RESULTS

The sample of 547 participants presented the following characteristics: 54.1% were women; the mean age was 76.2 years (±9.52); 68.9% were White; 44.1% were married; and 45.5% had an income between one and three minimum wages. Regarding frailty status, 46.07% were prefrail, 36.74% were frail, and 17.19% were non-frail. Among the frail older adults, 55.2% were female, the mean age was 80.9 (±9.5) years, 71.6% were White, 49.3% were widowed, 46.8% had an income between one and three minimum wages, and 74.7% had a low educational attainment (Table 1).

**Table 1:** Frequency distribution of sociodemographic characteristics and physical frailty status (N = 547), Curitiba, Paraná, Brazil, 2023

	Non-frail		Prefrail		Frail	
	(n = 94)	95%CI	(n = 252)	95%CI	(n = 201)	95%CI
Sex						
Female	45	47.9% (38.1-57.9)	140	55.6% (49.4-61.6)	111	55.2% (48.3-61.9)
Male	49	52.1% (42.1-61.9)	112	44.4% (38.4-50.6)	90	44.8% (38.1-51.7)
Age						
Mean (SD)	70.9 (7.26)		74.5 (8.62)		80.9 (9.5)	
Median [Min., Max.]	70 [60. 86]		74 [60. 98]		80 [61. 101]	
Race/Skin color						
White	63	67% (57-75.7)	170	67.5% (61.5-72.9)	144	71.6% (65.1-77.4)
Brown	27	28.7% (20.6-38.6)	65	25.8% (20.8-31.5)	44	21.9% (16.7-28.1)
Black	4	4.3% (1.7-10.4)	13	5.2% (3-8.6)	13	6.5% (3.8-10.7)
Other (Asian, Indigenous)	0	0% (0-3.9)	4	1.6% (0.6-4)	0	0% (0-1.9)
Marital status						
Married/Common-law marriage	50	53.2% (43.2-63)	118	46.8% (40.8-53)	73	36.3% (30-43.2)
Widowed	22	23.4% (16-32.9)	92	36.5% (30.8-42.6)	99	49.3% (42.4-56.1)
Divorced	15	16% (9.9-24.7)	29	11.5% (8.1-16)	19	9.5% (6.1-14.3)
Never married	7	7.4% (3.7-14.6)	12	4.8% (2.7-8.1)	8	4% (2-7.7)
Other	0	0% (0-3.9)	1	0.4% (0.1-2.2)	2	1% (0.3-3.6)
Educational attainment						
Higher education	7	7.4% (3.7-14.6)	14	5.6% (3.3-9.1)	10	5% (2.7-8.9)
High school	19	20.2% (13.3-29.4)	25	9.9% (6.8-14.2)	14	7% (4.2-11.4)
6th to 9th grade	14	14.9% (9.1-23.5)	50	19.8% (15.4-25.2)	27	13.4% (9.4-18.8)
1st to 5th grade	37	39.4% (30.1-49.5)	91	36.1% (30.4-42.2)	95	47.3% (40.5-54.2)
Literate (no formal schooling)	5	5.3% (2.3-11.9)	21	8.3% (5.5-12.4)	14	7% (4.2-11.4)
Illiterate	12	12.8% (7.5-21)	51	20.2% (15.7-25.6)	41	20.4% (15.4-26.5)
Living arrangement						
With family members	72	76.6% (67.9-84.7)	204	81%(75.7-85.3)	171	85.1% (80-89.7)
With caregiver	0	0% (0-4)	1	0.4% (0.1-2.2)	1	0.5% (0.1-2.8)
Long-term care facility	1	1.1% (0.2-5.8)	1	0.4% (0.1-2.2)	12	6% (3.5-10.2)
Lives alone	20	21.3% (14.4-30.9)	46	18.3% (14.0-23.5)	16	8% (5-12.6)
Missing data	1	(1.1%)	0	(0%)	1	(0.5%)
Income (Older adult)						
0-1	27	28.7% (20.6-38.6)	66	26.2% (21.2-32.1)	64	31.8% (25.9-38.8)
1,1-3	38	40.4% (31.1-50.5)	117	46.4% (40.5-52.8)	94	46.8% (40.2-53.9)
3,1-5	21	22.3% (15.1-31.8)	39	15.5% (11.6-20.5)	20	10% (6.6-14.9)
5,1-10	1	1.1% (0.2-5.8)	2	0.8% (0.2-2.9)	2	1% (0.3-3.6)
Over 10	0	0% (0-3.9)	0	0% (0-1.5)	0	0% (0-1.9)
No income	5	5.3% (2.3-11.9)	15	6% (3.7-9.6)	5	2.5% (1.1-5.7)
Did not report	2	2.1% (0.6-7.4)	12	4.8% (2.8-8.2)	15	7.5% (4.6-12)
Missing data	0	(0%)	1	(0.4%)	1	(0.5%)

*The minimum wage in effect during the data collection period was R$1,212.00, in accordance with Law No. 14,358/2022.*

Table 2 shows a progressive increase in the frequency distribution of clinical conditions according to frailty status (non-frail, prefrail, and frail). Noncommunicable diseases (NCDs)-such as previous stroke (9.6%, 19%, and 28.4%, respectively), dementia (7.4%, 12.7%, and 39.3%, respectively), chronic kidney disease (11.7%, 14.7%, and 19.9%, respectively), and chronic obstructive pulmonary disease (7.4%, 16.3%, and 18.4%, respectively)-were more frequent as frailty status progressed from non-frail to frail.

**Table 2:** Frequency distribution of clinical variables and physical frailty status (N = 547), Curitiba, Paraná, Brazil, 2023

	Non-frail	Prefrail	Frail
	(n = 94)	(n = 252)	(n = 201)
Comorbidities			
Asthma	3 (3.2%)	14 (5.6%)	11 (5.5%)
Stroke	9 (9.6%)	48 (19%)	57 (28.4%)
Cancer	10 (10.6%)	19 (7.5%)	16 (8%)
Dementia	7 (7.4%)	32 (12.7%)	79 (39.3%)
Depression	11 (11.7%)	31 (12.3%)	31 (15.4%)
*Diabetes mellitus*	33 (35.1%)	92 (36.5%)	79 (39.3%)
Dyslipidemia	33 (35.1%)	74 (29.4%)	62 (30.8%)
Parkinson’s disease	2 (2.1%)	10 (4%)	8 (4%)
Chronic kidney disease	11 (11.7%)	37 (14.7%)	40 (19.9%)
COPD	7 (7.4%)	41 (16.3%)	37 (18.4%)
Epilepsy	2 (2.1%)	9 (3.6%)	11 (5.5%)
BPH	11 (11.7%)	29 (11.5%)	26 (12.9%)
Hypertension	65 (69.1%)	193 (76.6%)	145 (72.1%)
Hypothyroidism	19 (20.2%)	47 (18.7%)	32 (15.9%)
CHF	9 (9.6%)	46 (18.3%)	33 (16.4%)

*COPD - chronic obstructive pulmonary disease; BPH - benign prostatic hyperplasia; CHF - congestive heart failure.*

As shown in Table 3, individuals classified as frail presented lower scores across all categories of the Perme Intensive Care Unit Mobility Score compared with non-frail and prefrail individuals.

**Table 3:** Distribution of Perme Intensive Care Unit Mobility Score categories and physical frailty status (N = 547), Curitiba, Paraná, Brazil, 2023

	Non-frail	Prefrail	Frail
	(n = 94)	(n = 252)	(n = 201)
Perme (total)			
Mean (SD)	27.6 (5.05)	25.9 (6.45)	15.2 (8.95)
Median [Min., Max.]	30 [10. 31]	29 [4. 31]	14 [0. 31]
Missing data	7 (7.4%)	4 (1.6%)	2 (1%)
Mental status			
Mean (SD)	3 (0)	2.95 (0.438)	2.48 (0.858)
Median [Min., Max.]	3 [3. 3]	3 [1. 8]	3 [0. 3]
Missing data	7 (7.4%)	4 (1.6%)	2 (1%)
Potential mobility barriers			
Mean (SD)	2.84 (0.428)	2.82 (0.477)	2.49 (0.635)
Median [Min., Max.]	3 [2. 4]	3 [0. 4]	3 [0. 4]
Missing data	7 (7.4%)	4 (1.6%)	2 (1%)
Functional strength			
Mean (SD)	3.98 (0.151)	3.85 (0.624)	3.01 (1.51)
Median [Min., Max.]	4 [3. 4]	4 [0. 5]	4 [0. 5]
Missing data	7 (7.4%)	4 (1.6%)	2 (1%)
Bed mobility			
Mean (SD)	5.77 (0.773)	5.37 (1.36)	3.10 (2.46)
Median [Min., Max.]	6 [0. 6]	6 [0. 6]	4 [0. 6]
Missing data	7 (7.4%)	4 (1.6%)	3 (1.5%)
Transfers			
Mean (SD)	7.94 (2.54)	7.34 (2.97)	3.06 (3.77)
Median [Min., Max.]	9 [0. 9]	9 [0. 9]	0 [0. 9]
Missing data	7 (7.4%)	4 (1.6%)	7 (3.5%)
Gait			
Mean (SD)	2.39 (1.11)	2.11 (1.23)	0.608 (1.08)
Median [Min., Max.]	3 [0. 3]	3 [0. 3]	0 [0. 3]
Missing data	9 (9.6%)	7 (2.8%)	7 (3.5%)
Endurance			
Mean (SD)	1.88 (1.17)	1.58 (1.20)	0.412 (0.843)
Median [Min., Max.]	2 [0. 3]	1 [0. 3]	0 [0. 3]
Missing data	9 (9.6%)	7 (2.8%)	7 (3.5%)

*SD - standard deviation.*

Table 4 shows a statistically significant difference in both the total Perme Score and its individual categories between frail and non-frail participants (p < 0.001) and between frail and prefrail participants (p < 0.001). No statistically significant difference was observed between prefrail and non-frail participants (p = 0.1409).

**Table 4:** Relationship between the total and mean scores of the Perme Intensive Care Unit Mobility Score categories and physical frailty status (N = 547), Curitiba, Paraná, Brazil, 2023

	Difference	95% CI	*p* value
Perme (total)			
Prefrail vs. Non-frail	-1.73	-3.86; 0.41	0.1409
Frail vs. Non-frail	-12.45	-14.66; -10.24	< 0.001
Frail vs. Prefrail	-10.72	-12.36; -9.09	< 0.001
Mental status			
Prefrail vs. Non-frail	-0.05	-0.22; 0.13	0.7959
Frail vs. Non-frail	-0.52	-0.7; -0.34	< 0.001
Frail vs. Prefrail	-0.47	-0.6; -0.33	< 0.001
Potential mobility barriers			
Prefrail vs. Non-frail	-0.02	-0.17; 0.14	0.9667
Frail vs. Non-frail	-0.35	-0.51; -0.19	< 0.001
Frail vs. Prefrail	-0.33	-0.45; -0.21	< 0.001
Functional strength			
Prefrail vs. Non-frail	-0.13	-0.43; 0.17	0.5584
Frail vs. Non-frail	-0.97	-1.28; -0.67	< 0.001
Frail vs. Prefrail	-0.84	-1.07; -0.61	< 0.001
Bed mobility			
Prefrail vs. Non-frail	-0.40	-0.93; 0.12	0.1689
Frail vs. Non-frail	-2.67	-3.21; -2.13	< 0.001
Frail vs. Prefrail	-2.27	-2.67; -1.86	< 0.001
Transfers			
Prefrail vs. Non-frail	-0.60	-1.55; 0.34	0.2908
Frail vs. Non-frail	-4.88	-5.86; -3.9	< 0.001
Frail vs. Prefrail	-4.28	-5; -3.55	< 0.001
Gait			
Prefrail vs. Non-frail	-0.27	-0.62; 0.07	0.1443
Frail vs. Non-frail	-1.78	-2.13; -1.43	< 0.001
Frail vs. Prefrail	-1.51	-1.77; -1.25	< 0.001
Endurance			
Prefrail vs. Non-frail	-0.30	-0.62; 0.02	0.0713
Frail vs. Non-frail	-1.47	-1.8; -1.14	< 0.001
Frail vs. Prefrail	-1.17	-1.41; -0.93	< 0.001

*Tukey test; significant p-value (p ≤ 0.05); hypothesis test for equality of proportions.*

## DISCUSSION

The predominance of sociodemographic characteristics in the sample is consistent with data from the 2022 Brazilian Census, which show that the municipality of Curitiba (PR) has a majority female population (52.81%), predominantly White individuals (74.44%), and an average income of 3.7 minimum wages^(25)^.

The high prevalence of prefrail (46.97%) and frail (36.74%) older adults identified in this study differs from findings in a systematic review on frailty in this age group. The review included 62 studies in the prefrailty analysis, revealing a prevalence of 25.8% (95% CI: 22%-29.6%); for frailty, 96 studies were analyzed, with a total sample of 467,779 participants and a prevalence of 47.4% (95% CI: 43.7%-51.1%)^(26)^. When only the studies that used the same frailty assessment instrument as in the present study were considered, the global prevalence of frailty ranged from 47.4% (95% CI: 43.7%-51.1%) to 42.9% (95% CI: 35.4%-50.4%)^(26)^. In addition to differences in the instruments used to assess frailty, a possible explanation for this reversed prevalence may involve sex-related frailty patterns, the inclusion of individuals aged 65 and older, and the diversity of study settings.

Conversely, studies conducted in Curitiba (PR) with community-dwelling and hospitalized older adults identified a predominance of the prefrail condition. A cross-sectional study conducted with a community-based sample of 1,716 participants aged 60 to 96 years (mean age 71 ± 7.3) found that 65.3% were prefrail and 15.8% were frail^(27)^. Another cross-sectional study involving 320 hospitalized individuals aged 60 years or older identified 49% as prefrail and 36.2% as frail^(28)^.

The frequency distribution of clinical conditions-such as stroke, dementia, cardiovascular disease, and chronic kidney disease-was directly proportional to the frailty status. Supporting these findings, a study conducted in China that included 7,116 participants from the China Health and Retirement Longitudinal Study (CHARLS), 5,303 from the English Longitudinal Study of Ageing (ELSA), and 7,266 from the Health and Retirement Study (HRS) showed that robust participants who progressed to a prefrail or frail state had increased risks of developing cardiovascular disease (CHARLS, HR 1.84; 95% CI: 1.54-2.21; ELSA, HR 1.53; 95% CI: 1.25-1.86; and HRS, HR 1.59; 95% CI: 1.31-1.92)^(29)^.

In the present study, non-frail patients demonstrated higher mobility levels (Perme score of 27.6), followed by prefrail patients (Perme score of 25.9) and frail patients (Perme score of 15.2). However, no studies were found in the literature that explored this relationship between the Perme score and frailty status. Existing studies focus solely on mobility assessment using the Perme score or on associations between the Perme score and specific clinical conditions, such as liver transplantation^(30)^, cardiac surgery^(31)^, tracheostomy^(32)^, and organophosphate poisoning^(33)^.

Most of these studies were conducted in intensive care units, where ambulation is more restricted, and aimed to determine patients’ mobility scores. One example is a methodological study conducted with 50 patients in a coronary ICU in Turkey, with a mean age of 69 years. The results showed a mean Perme score of 22 (±5)^(34)^, a value close to the mean score found in the present study (22.9), which, however, was conducted in the medical wards of a hospital specialized in the care of older adults.

Another study, conducted outside the ICU setting, used a retrospective cohort of 69 patients with COVID-19 to examine changes in mobility during hospitalization. The mean Perme score at admission to the ward was 17.5 (95% CI: 15.8-19.3), which increased to 24.8 (95% CI: 23.3-26.3) at discharge. An average increase of 7.3 points (95% CI: 5.7-8.8; p < 0.001) in mobility was observed throughout hospitalization, indicating a significant improvement between admission and discharge^(35)^. It is important to note, however, that these patients had COVID-19, a condition with distinct clinical characteristics that differ significantly from those of other causes of hospitalization.

Although most studies apply the Perme score in ICUs, some have used it in other care settings and clinical contexts^(30,35)^. This finding demonstrates the potential of the tool for mobility assessment across different healthcare environments and conditions, as well as for tracking mobility progression during hospitalization. Early mobilization should always be encouraged, while respecting each patient’s contraindications and limitations.

Impaired mobility contributes to the frailty process and increases the risk of adverse events during hospitalization^(18,19)^. Although mobility impairment is common, neither mobility nor balance dysfunction alone is sufficient to define frailty. Mobility should be routinely assessed, as many older adults experience a decline in physical conditioning during hospitalization. Furthermore, regular mobility assessments should be carried out to monitor disease progression and recovery, especially in the oldest patients. These issues are becoming increasingly important due to the growing demand for hospitalization among older adults, particularly those who are frail.

The findings of this study show that hospitalized frail older adults exhibit significantly reduced mobility. This is in line with international guidelines that identify loss of muscle strength and function as core components of frailty^(17)^. These results underscore the importance of early identification and intervention in prefrail patients, as they maintain mobility levels comparable to those of non-frail individuals, suggesting a window of opportunity for prevention. However, the hospital environment presents additional challenges, such as barriers to mobilization and limitations of assessment tools, which point to the need for adapting international recommendations to clinical practice in medical wards. In this context, integrating systematic screening with adapted mobilization protocols may improve the functional prognosis of these patients.

The use of the Perme score is valuable as a tool for assessing mobility across different healthcare settings and clinical conditions. It is also useful for tracking mobility progression throughout hospitalization.

### Study limitations

A key limitation of this study lies in the gap in the literature regarding research analyzing the relationship between frailty and mobility. This shortcoming restricted the discussion of findings related to these variables.

Information bias was minimized through a rigorous study design, interviewer training, calibration of data collection instruments, and support from a statistics expert during the data analysis and interpretation phases. It was also mitigated by the development of a standardized protocol for data collection, measurement, and interpretation. To address the literature gap, a scoping review was conducted on the Perme mobility assessment instrument.

### Contributions to the field

The findings of this study may contribute to improving the management of care for hospitalized older adults, as the data highlight the importance of care grounded in systematic assessment and early mobilization during hospitalization. Such care management becomes more effective when led by a multidisciplinary team that remains attentive to factors associated with reduced mobility, among which physical frailty stands out, as it is frequently linked to adverse outcomes during hospitalization.

## CONCLUSIONS

Frail older adults hospitalized in inpatient wards demonstrated lower mobility compared to non-frail and prefrail individuals. This association was not observed when comparing non-frail and prefrail participants.

The reduced mobility among frail hospitalized older adults highlights the need to develop early mobilization protocols. This is particularly important because mobility limitations contribute to physical frailty, which in turn leads to functional decline and disability in this population.

## Data Availability

The research data are available in a repository: https://doi.org/10.48331/scielodata.MTY89M.
