# Supplementary material for: Frailty and mobility in hospitalized older adults: an analytical cross sectional study
Source: Rev Bras Enferm. 2025 Nov 3;78(Suppl 2):e20240570. doi: 10.1590/0034-7167-2024-0570 (PMC12584953; doi:10.1590/0034-7167-2024-0570)
Supplement: Translation

# Translation: Fragilidade e mobilidade física na pessoa idosa hospitalizada: estudo transversal analítico

**DOI:** 10.1590/0034-7167-2024-0570pt

**Authors:** Maria Helena Lenardt, Clovis Cechinel, João Alberto Martins Rodrigues, Tissiane Bona Zomer, Maria Angélica Binotto, Larissa Teleginski Wardenski

**Affiliations:** I Universidade Federal do Paraná. Curitiba, Paraná, Brasil; II Fundação Estatal de Atenção à Saúde. Curitiba, Paraná, Brasil

**Keywords:** Deambulação Precoce, Fragilidade, Hospitalização, Idoso, Limitação da Mobilidade.

## Abstract

**Objetivos:**

analisar a relação entre a condição de fragilidade e mobilidade física na pessoa idosa hospitalizada em enfermaria.

**Métodos:**

estudo transversal analítico, conduzido em hospital do Sul do Brasil. Para obtenção dos dados, utilizou-se questionário de informações sociodemográficas e clínicas, marcadores do fenótipo de fragilidade física e o *Perme Intensive Care Unit Mobility Score.* Incluíram-se pacientes com 60 anos ou mais, hospitalizados em enfermarias. Excluíram-se casos críticos, com indicação de UTI, aguardando transferência ou em precaução de gotículas. Empregou se estatística descritiva, teste de Tukey e teste de hipótese de igualdade das proporções.

**Resultados:**

dos 547 participantes, 46,07% eram pré-frágeis e 36,74% frágeis. Houve associação dos escores de mobilidade física entre pessoas idosas frágeis 15,2 (±8,95) e não frágeis 27,6 (±5,05), *p*<0,001 e entre frágeis 15,2 (±8,95) e pré-frágeis 25,9 (±6,45), *p*<0,001.

**Conclusões:**

pessoas idosas frágeis hospitalizadas em enfermaria apresentaram menor mobilidade quando comparadas às não frágeis e pré-frágeis.

## INTRODUÇÃO

No processo de envelhecimento, os músculos, ossos e articulações sofrem alterações fisiológicas que podem afetar a mobilidade física^(1)^ e, assim, impactar a independência de quem vivencia esse processo. Por apresentar uma coleção de sinais e sintomas, a mobilidade física em pessoas idosas classifica-se como uma síndrome geriátrica^(2)^. Ela é um marcador e preditor de habilidade física, independência, morbidade e mortalidade na população idosa^(3)^.

Por ser uma síndrome geriátrica de prevalência elevada, o diagnóstico, avaliação e tratamento da limitação de mobilidade são fundamentais. As limitações devido à redução da massa óssea e muscular^(1)^ foram objeto de diversas revisões de literatura com o propósito de analisar as associações com outras variáveis, tais como sexo feminino^(4,5)^, baixa atividade física^(5,6)^, nível socioeconômico, estado nutricional^(7)^ e deficiências sensoriais, cognitivas e físicas^(4-6)^. No entanto, essas revisões centraram-se principalmente nas populações idosas residentes na comunidade.

Ademais, nas análises sobre as condições clínicas, a maior parte dos estudos publicados avalia a relação da limitação da mobilidade em pessoas idosas com multimorbidade^(8,9)^ e doenças crônicas não transmissíveis, como as seguintes: doenças neurológicas^(10)^, cardíacas^(11)^ e respiratórias^(12)^, depressão^(13)^, diabetes e osteoartrite^(6)^, acidente vascular encefálico^(14)^ e demências^(4,15,16)^.

A associação entre mobilidade e fragilidade física durante a hospitalização de pessoas idosas não foi suficientemente explorada em estudos nacionais e internacionais. A fragilidade física está entre os principais determinantes do declínio funcional e mortalidade precoce nesse segmento etário, já que se caracteriza como estado clínico identificado por aumento da vulnerabilidade no indivíduo quando exposto a estressores internos e externos^(17)^.

O declínio na mobilidade ocorre em doenças agudas e hospitalizações e, nesse cenário, pode ser redefinido como estar restrito ao leito ou transferir-se apenas da cama para a cadeira, com consequente redução de caminhada durante a hospitalização^(2)^. Os declínios funcionais associados à hospitalização em pacientes mais velhos prolongam a permanência hospitalar e aumentam a carga de cuidados pós-alta, risco de incapacidade, mortalidade e despesas médicas^(18,19)^.

Considerando que a hospitalização está associada a declínio adicional na capacidade funcional, acredita-se que os resultados deste estudo suscitem intervenções para mitigar esse declínio na pessoa idosa com fragilidade física.

## OBJETIVOS

Analisar a relação entre a condição de fragilidade física e mobilidade física na pessoa idosa hospitalizada em enfermaria.

## MÉTODOS

O relatório do estudo seguiu as diretrizes do checklist *STrengthening the Reporting of OBservational studies in Epidemiology* (STROBE)^(20)^.

### Desenho e local do estudo

Trata-se de estudo de corte transversal analítico, realizado com pessoas idosas avaliadas nas primeiras 48 horas da admissão nas enfermarias de um hospital de média complexidade e referência para idosos localizado na Região Sul do Brasil.

### População, Critérios de Inclusão e Exclusão

A população do estudo foi composta por idosos hospitalizados em enfermaria. Incluíram-se indivíduos com idade igual ou superior a 60 anos, hospitalizados em enfermarias clínicas ou cirúrgicas. Foram excluídos indivíduos com quadro crítico, como indicação de internação em UTI aguardando transferência, ou que estivessem em precaução de gotículas.

### Cálculo da Amostra e Coleta de Dados

Para determinar o tamanho da amostra, utilizou-se prevalência de 50%, visando garantir o maior número possível de participantes. Utilizou-se cálculo amostral para população com idade de tamanho conhecido, representativa dos internamentos no ano 2019 (n = 4.146). Definiu-se nível de confiança de 95% e erro amostral de 5%. Com base nesses parâmetros, o tamanho amostral mínimo calculado foi de 352 indivíduos. Neste estudo, adotou-se uma amostra de 547 participantes, a fim de atingir erro amostral inferior a 5%.

Antes da aplicação dos testes, realizaram-se treinamentos teóricos e práticos com integrantes de um grupo de pesquisa composto por estudantes de graduação, residentes multiprofissionais e pós graduandos de uma instituição federal de ensino, com objetivo de uniformizar a coleta e diminuir o risco de possíveis vieses. A coleta de dados ocorreu entre março de 2022 e julho de 2023, nas enfermarias do hospital. O tempo médio de aplicação dos instrumentos de coleta foi de cerca de 30 minutos.

Primeiramente, realizou-se rastreio cognitivo utilizando-se o Miniexame do Estado Mental^(21)^, validado para uso em português do Brasil^(22)^, a fim de selecionar o segmento etário idoso apto para responder os questionários e assinar o Termo de Consentimento Livre e Esclarecido (TCLE). Quando a pessoa idosa apresentava alteração cognitiva e/ou déficit de comunicação, o acompanhante era convidado exclusivamente para responder os itens dos questionários e assinar o TCLE, sendo que os testes foram aplicados na pessoa idosa. Se o acompanhante presente no momento da aplicação dos questionários não se julgasse apto a responder às questões, o acompanhante principal era acionado para garantir a veracidade das informações. Neste estudo, considerou-se acompanhante principal o sujeito que presta cuidados diretos à pessoa idosa e convive com ela há, pelo menos, três meses.

A coleta de dados teve início com a aplicação do questionário sociodemográfico e clínico, incluindo as seguintes variáveis de interesse para a pesquisa: sociodemográficas - idade, sexo, cor, estado civil, escolaridade, com quem reside e renda; e clínicas - asma, acidente vascular encefálico (AVE), câncer, demência, depressão, diabetes *mellitus*, dislipidemia, doença de Parkinson, doença renal crônica (DRC), doença pulmonar obstrutiva crônica (DPOC), epilepsia, hiperplasia de próstata benigna (HPB), hipertensão arterial, hipotireoidismo e insuficiência cardíaca (IC).

A condição de fragilidade física foi classificada mediante os marcadores do fenótipo de fragilidade física, redução da força de preensão manual, diminuição da velocidade da marcha, perda de peso não intencional, redução do nível de atividade física e autorrelato de fadiga/exaustão. São considerados pré-frágeis os participantes com um ou dois marcadores, e frágeis aqueles com três ou mais. Os participantes que possuem um ou dois componentes são caracterizados como pré-frágeis e aqueles com três ou mais são considerados frágeis^(23)^.

Obteve-se o escore da mobilidade dos participantes mediante busca no prontuário eletrônico de registros do fisioterapeuta no dia da coleta dos demais dados. O *Perme Intensive Care Unit Mobility Score* fornece uma pontuação total (0 a 32 pontos) que avalia o status de mobilidade do paciente.

O escore *Perme* avalia a mobilidade física por meio de sete categorias principais.

- A primeira refere se ao estado mental, considera o nível de alerta e a capacidade de obedecer a comandos.
- A segunda categoria aborda potenciais barreiras para a mobilidade, como o uso de ventilação mecânica invasiva ou não invasiva, presença de dor, acessos venosos, cateteres, sonda nasogástrica e drenos torácicos.
- A terceira avalia a força funcional, verificando a capacidade do paciente de realizar flexão de ombro em pelo menos 45° e flexão de quadril em pelo menos 20°.
- A quarta categoria trata da mobilidade no leito, incluindo a mudança da posição supina para sentada e o equilíbrio estático nessa posição à beira do leito, podendo haver auxílio do terapeuta em diferentes níveis.
- A quinta envolve a transferência e analisa a capacidade de mudar da posição sentada para em pé, manter o equilíbrio estático e realizar transferências entre leito e cadeira.
- A sexta categoria avalia a marcha, observando a capacidade de deambular com ou sem dispositivos de auxílio e com diferentes níveis de assistência do terapeuta.
- Por fim, a sétima categoria mede a *endurance*, correspondendo à distância percorrida durante a deambulação em dois minutos, com ou sem dispositivos de auxílio^(24)^.

### Análise e Tratamento dos Dados

Aplicaram-se métodos de estatística descritiva para analisar as características sociodemográficas em relação à condição de fragilidade. Efetuaram-se cálculos de médias, desvios padrão, medianas, máximos, mínimos e tabelas de frequência. O teste de Tukey foi empregado na comparação de médias para os valores dos índices de *Perme* e seus subíndices considerando a condição de fragilidade. Para comparar as proporções dos índices de *Perme* para as diferentes condições de fragilidade, foi utilizado o teste de hipótese de igualdade das proporções. Todos os testes de hipóteses foram realizados com 5% de significância. Executaram-se as análises estatísticas no software R 4.2.2.

### Aspectos éticos

O presente estudo bderiva do estudo-matriz “Fragilidade física e os desfechos clínicos, funcionais, psicossociais, nutricionais e na demanda de cuidados em idosos hospitalizados”. Foi aprovado pelo Comitê de Ética em Pesquisa em Seres Humanos do Setor de Ciências da Saúde da Universidade Federal do Paraná e pelo Comitê de Ética em Pesquisa em Seres Humanos da Secretaria Municipal de Saúde de Curitiba. O consentimento formal, por meio do Termo de Consentimento Livre e Esclarecido, foi obtido da pessoa idosa e/ou acompanhante antes do início do estudo, conforme a Resolução 466/12. Da mesma forma, garantiu-se o sigilo e a confidencialidade dos dados de acordo com o disposto nas Resoluções nº 510/2016, nº 580/2018 e demais resoluções do Conselho Nacional de Saúde/MS vigentes na época do estudo.

## RESULTADOS

A amostra de 547 participantes apresentou as seguintes características: 54,1% eram mulheres, a média de idade era de 76,2 anos (± 9,52), 68,9% tinham a cor da pele branca, 44,1% eram casadas e 45,5% tinham renda entre um e três salários mínimos. Quanto à condição de fragilidade, 46,07% eram pré-frágeis, 36,74% frágeis e 17,19% não frágeis. Das pessoas idosas na condição de frágeis, 55,2% eram do sexo feminino, a média de idade era de 80,9 (± 9,5) anos, 71,6% eram brancas, 49,3% eram viúvas, 46,8% tinham com renda de um a três salários mínimos e 74,7% apresentavam baixa escolaridade (Tabela 1).

**Tabela 1:** Distribuição de frequência das características sociodemográficas e condição de fragilidade física (N = 547), Curitiba, Paraná, Brasil, 2023

	Não frágil		Pré-frágil		Frágil	
	(n = 94)	IC 95%	(n = 252)	IC 95%	(n = 201)	IC 95%
Sexo						
Feminino	45	47,9% (38,1-57,9)	140	55,6% (49,4-61,6)	111	55,2% (48,3-61,9)
Masculino	49	52,1% (42,1-61,9)	112	44,4% (38,4-50,6)	90	44,8% (38,1-51,7)
Idade						
Média (DP)	70,9 (7,26)		74,5 (8,62)		80,9 (9,5)	
Mediana [Mín., Máx.]	70 [60, 86]		74 [60, 98]		80 [61, 101]	
Raça/Cor						
Branca	63	67% (57-75,7)	170	67,5% (61,5-72,9)	144	71,6% (65,1-77,4)
Parda	27	28,7% (20,6-38,6)	65	25,8% (20,8-31,5)	44	21,9% (16,7-28,1)
Preta	4	4,3% (1,7-10,4)	13	5,2% (3-8,6)	13	6,5% (3,8-10,7)
Outros (orientais, indígenas)	0	0% (0-3,9)	4	1,6% (0,6-4)	0	0% (0-1,9)
Estado civil						
Casado(a)/União estável	50	53,2% (43,2-63)	118	46,8% (40,8-53)	73	36,3% (30-43,2)
Viúvo(a)	22	23,4% (16-32,9)	92	36,5% (30,8-42,6)	99	49,3% (42,4-56,1)
Divorciado(a)	15	16% (9,9-24,7)	29	11,5% (8,1-16)	19	9,5% (6,1-14,3)
Solteiro(a)	7	7,4% (3,7-14,6)	12	4,8% (2,7-8,1)	8	4% (2-7,7)
Outros	0	0% (0-3,9)	1	0,4% (0,1-2,2)	2	1% (0,3-3,6)
Escolaridade						
Ensino superior	7	7,4% (3,7-14,6)	14	5,6% (3,3-9,1)	10	5% (2,7-8,9)
Ensino médio	19	20,2% (13,3-29,4)	25	9,9% (6,8-14,2)	14	7% (4,2-11,4)
6º a 9º ano	14	14,9% (9,1-23,5)	50	19,8% (15,4-25,2)	27	13,4% (9,4-18,8)
1º a 5º ano	37	39,4% (30,1-49,5)	91	36,1% (30,4-42,2)	95	47,3% (40,5-54,2)
Lê e escreve	5	5,3% (2,3-11,9)	21	8,3% (5,5-12,4)	14	7% (4,2-11,4)
Analfabeto	12	12,8% (7,5-21)	51	20,2% (15,7-25,6)	41	20,4% (15,4-26,5)
Com quem reside						
Familiares	72	76,6% (67,9-84,7)	204	81%(75,7-85,3)	171	85,1% (80-89,7)
Cuidador(a)	0	0% (0-4)	1	0,4% (0,1-2,2)	1	0,5% (0,1-2,8)
ILPI	1	1,1% (0,2-5,8)	1	0,4% (0,1-2,2)	12	6% (3,5-10,2)
Sozinho(a)	20	21,3% (14,4-30,9)	46	18,3% (14,0-23,5)	16	8% (5-12,6)
Faltantes	1	(1,1%)	0	(0%)	1	(0,5%)
Renda (Idoso)						
0 - 1	27	28,7% (20,6-38,6)	66	26,2% (21,2-32,1)	64	31,8% (25,9-38,8)
1,1 - 3	38	40,4% (31,1-50,5)	117	46,4% (40,5-52,8)	94	46,8% (40,2-53,9)
3,1 - 5	21	22,3% (15,1-31,8)	39	15,5% (11,6-20,5)	20	10% (6,6-14,9)
5,1 - 10	1	1,1% (0,2-5,8)	2	0,8% (0,2-2,9)	2	1% (0,3-3,6)
Acima de 10	0	0% (0-3,9)	0	0% (0-1,5)	0	0% (0-1,9)
Sem Renda	5	5,3% (2,3-11,9)	15	6% (3,7-9,6)	5	2,5% (1,1-5,7)
Não informou	2	2,1% (0,6-7,4)	12	4,8% (2,8-8,2)	15	7,5% (4,6-12)
Faltantes	0	(0%)	1	(0,4%)	1	(0,5%)

*O salário mínimo vigente no período de coleta do estudo era de R$ 1.212,00, conforme Lei nº 14.358/2022; ILPI - Instituição de Longa Permanência para Idosos.*

A Tabela 2 mostra um aumento crescente na distribuição de frequência das condições clínicas quando comparadas à condição de fragilidade (não frágil, pré-frágil e frágil). As doenças crônicas não transmissíveis (DCNT) - como acidente vascular encefálico prévio (9,6%; 19% e 28,4%, respectivamente), demência (7,4%; 12,7% e 39,3%, respectivamente), doença renal crônica (11,7%; 14,7% e 19,9%, respectivamente) e DPOC (7,4%; 16,3% e 18,4%, respectivamente) - apresentaram maior frequência à medida que houve transição do estado não frágil para o frágil.

**Tabela 2:** Distribuição de frequência das variáveis clínicas e condição de fragilidade física (N = 547), Curitiba, Paraná, Brasil, 2023

	Não frágil	Pré-frágil	Frágil
	(n = 94)	(n = 252)	(n = 201)
Morbidades			
Asma	3 (3,2%)	14 (5,6%)	11 (5,5%)
AVE	9 (9,6%)	48 (19%)	57 (28,4%)
Câncer	10 (10,6%)	19 (7,5%)	16 (8%)
Demência	7 (7,4%)	32 (12,7%)	79 (39,3%)
Depressão	11 (11,7%)	31 (12,3%)	31 (15,4%)
Diabetes *mellitus*	33 (35,1%)	92 (36,5%)	79 (39,3%)
Dislipidemia	33 (35,1%)	74 (29,4%)	62 (30,8%)
Doença de Parkinson	2 (2,1%)	10 (4%)	8 (4%)
Doença renal crônica	11 (11,7%)	37 (14,7%)	40 (19,9%)
DPOC	7 (7,4%)	41 (16,3%)	37 (18,4%)
Epilepsia	2 (2,1%)	9 (3,6%)	11 (5,5%)
HPB	11 (11,7%)	29 (11,5%)	26 (12,9%)
Hipertensão	65 (69,1%)	193 (76,6%)	145 (72,1%)
Hipotireoidismo	19 (20,2%)	47 (18,7%)	32 (15,9%)
ICC	9 (9,6%)	46 (18,3%)	33 (16,4%)

*AVE - acidente vascular encefálico; DPOC - doença pulmonar obstrutiva crônica; HBP - hiperplasia benigna de próstata; ICC - insuficiência cardíaca congestiva.*

Na Tabela 3, nota-se que indivíduos classificados como frágeis apresentaram valores menores para todas as categorias do *Perme Intensive Care Unit Mobility Score* quando comparados a indivíduos não frágeis ou pré-frágeis.

**Tabela 3:** Distribuição das categorias do *Perme Intensive Care Unit Mobility Score* e condição de fragilidade física (N = 547), Curitiba, Paraná, Brasil, 2023

	Não frágil	Pré-frágil	Frágil
	(n = 94)	(n = 252)	(n = 201)
*Perme* (total)			
Média (DP)	27,6 (5,05)	25,9 (6,45)	15,2 (8,95)
Mediana [Mín., Máx.]	30 [10, 31]	29 [4, 31]	14 [0, 31]
Faltantes	7 (7,4%)	4 (1,6%)	2 (1%)
Estado mental			
Média (DP)	3 (0)	2,95 (0,438)	2,48 (0,858)
Mediana [Mín., Máx.]	3 [3, 3]	3 [1, 8]	3 [0, 3]
Faltantes	7 (7,4%)	4 (1,6%)	2 (1%)
Potenciais barreiras para mobilidade			
Média (DP)	2,84 (0,428)	2,82 (0,477)	2,49 (0,635)
Mediana [Mín., Máx.]	3 [2, 4]	3 [0, 4]	3 [0, 4]
Faltantes	7 (7,4%)	4 (1,6%)	2 (1%)
Força funcional			
Média (DP)	3,98 (0,151)	3,85 (0,624)	3,01 (1,51)
Mediana [Mín., Máx.]	4 [3, 4]	4 [0, 5]	4 [0, 5]
Faltantes	7 (7,4%)	4 (1,6%)	2 (1%)
Mobilidade no leito			
Média (DP)	5,77 (0,773)	5,37 (1,36)	3,10 (2,46)
Mediana [Mín., Máx.]	6 [0, 6]	6 [0, 6]	4 [0, 6]
Faltantes	7 (7,4%)	4 (1,6%)	3 (1,5%)
Transferência			
Média (DP)	7,94 (2,54)	7,34 (2,97)	3,06 (3,77)
Mediana [Mín., Máx.]	9 [0, 9]	9 [0, 9]	0 [0, 9]
Faltantes	7 (7,4%)	4 (1,6%)	7 (3,5%)
Marcha			
Média (DP)	2,39 (1,11)	2,11 (1,23)	0,608 (1,08)
Mediana [Mín., Máx.]	3 [0, 3]	3 [0, 3]	0 [0, 3]
Faltantes	9 (9,6%)	7 (2,8%)	7 (3,5%)
*Endurance*			
Média (DP)	1,88 (1,17)	1,58 (1,20)	0,412 (0,843)
Mediana [Mín., Máx.]	2 [0, 3]	1 [0, 3]	0 [0, 3]
Faltantes	9 (9,6%)	7 (2,8%)	7 (3,5%)

*DP - desvio-padrão.*

Na Tabela 4, observa-se a diferença significante entre os valores do *Perme Intensive Care Unit Mobility Score* tanto no valor total quanto em suas categorias, entre os participantes frágeis e não frágeis (p < 0,001) e frágeis e pré-frágeis (p < 0,001). Não houve diferença estatisticamente significante na comparação entre pré-frágeis e não frágeis (p = 0,1409).

**Tabela 4:** Relação entre o total e as médias das categorias do *Perme Intensive Care Unit Mobility Score* e a condição de fragilidade física (N = 547), Curitiba, Paraná, Brasil, 2023

	Diferença	IC	Valor de *p*
Total			
Pré-frágil-Não frágil	-1,73	-3,86; 0,41	0,1409
Frágil-Não frágil	-12,45	-14,66; -10,24	< 0,001
Frágil-Pré-frágil	-10,72	-12,36; -9,09	< 0,001
Estado mental			
Pré-frágil-Não frágil	-0,05	-0,22; 0,13	0,7959
Frágil-Não frágil	-0,52	-0,7; -0,34	< 0,001
Frágil-Pré-frágil	-0,47	-0,6; -0,33	< 0,001
Potenciais barreiras para mobilidade			
Pré-frágil-Não frágil	-0,02	-0,17; 0,14	0,9667
Frágil-Não frágil	-0,35	-0,51; -0,19	< 0,001
Frágil-Pré-frágil	-0,33	-0,45; -0,21	< 0,001
Força funcional			
Pré-frágil-Não frágil	-0,13	-0,43; 0,17	0,5584
Frágil-Não frágil	-0,97	-1,28; -0,67	< 0,001
Frágil-Pré-frágil	-0,84	-1,07; -0,61	< 0,001
Mobilidade no leito			
Pré-frágil-Não frágil	-0,40	-0,93; 0,12	0,1689
Frágil-Não frágil	-2,67	-3,21; -2,13	< 0,001
Frágil-Pré-frágil	-2,27	-2,67; -1,86	< 0,001
Transferência			
Pré-frágil-Não frágil	-0,60	-1,55; 0,34	0,2908
Frágil-Não frágil	-4,88	-5,86; -3,9	< 0,001
Frágil-Pré-frágil	-4,28	-5; -3,55	< 0,001
Marcha			
Pré-frágil-Não frágil	-0,27	-0,62; 0,07	0,1443
Frágil-Não frágil	-1,78	-2,13; -1,43	< 0,001
Frágil-Pré-frágil	-1,51	-1,77; -1,25	< 0,001
Endurance			
Pré-frágil-Não frágil	-0,30	-0,62; 0,02	0,0713
Frágil-Não frágil	-1,47	-1,8; -1,14	< 0,001
Frágil-Pré-frágil	-1,17	-1,41; -0,93	< 0,001

*Teste de Tukey; valor de p significante (p ≤ 0,05); teste de hipótese de igualdade das proporções.*

## DISCUSSÃO

O predomínio das características sociodemográficas da amostra é corroborado nas informações coletadas no último censo do IBGE de 2022, as quais mostram que o município de Curitiba (PR) tem predomínio de mulheres (52,81%), indivíduos brancos (74,44%) e com renda média de 3,7 salários mínimos^(25)^.

A prevalência elevada de pessoas idosas pré-frágeis (46,97%) e frágeis (36,74%) identificada nesta pesquisa difere dos achados de revisão sistemática sobre fragilidade nesse grupo etário. Na revisão, incluíram-se na análise de pré-fragilidade 62 estudos, que evidenciaram prevalência de 25,8% (IC95% 22% a 29,6%); já na análise de fragilidade, foram 96 estudos, com amostra total de 467.779 participantes e taxa de 47,4% (IC95% 43,7% a 51,1%^(26)^. Ao serem considerados apenas os estudos que incluíram o mesmo instrumento de avaliação de fragilidade que utilizamos, observou-se variação da prevalência global de fragilidade de 47,4% (IC95% 43,7 a 51,1%) para 42,9% (IC95% 35,4% a 50,4%)^(26)^. Como uma possível explicação para a inversão de prevalência, além dos diferentes instrumentos utilizados para avaliação de fragilidade, destacam-se as questões de fragilidade relacionadas ao sexo, a inclusão de pacientes com idade superior a 65 anos e a diversidade de locais de realização dos estudos.

Em contrapartida, estudos desenvolvidos em Curitiba (PR) com pessoas idosas da comunidade hospitalizadas identificaram prevalência da condição de pré-fragilidade. O estudo transversal - conduzido com uma amostra de 1.716 participantes da comunidade na faixa etária de 60 a 96 anos e média de idade 71 (±7,3) - apontou 65,3% de pré-frágeis e 15,8% de frágeis^(27)^. Outro estudo transversal, realizado com 320 indivíduos hospitalizados e com idade maior ou igual a 60 anos, identificou 49% de pré-frágeis e 36,2% de frágeis^(28)^.

A distribuição de frequência das condições clínicas, AVE, demência, doença cardiovascular e doença renal crônica é diretamente proporcional à condição de fragilidade. Corrobora esses dados, um estudo conduzido na China, que incluiu 7.116 participantes do *China Health and Retirement Longitudinal Study* (CHARLS), 5.303 do *English Longitudinal Study of Ageing* (ELSA) e 7.266 do *Health and Retirement Study* (HRS). Os participantes robustos que progrediram para o estado pré frágil ou frágil apresentaram riscos aumentados de ocorrência de DCV (CHARLS, HR 1,84 e IC95%: 1,54-2,21; ELSA, HR 1,53 e IC95% 1,25-1,86; e HRS, HR 1,59 e IC95% 1,31-1,92)^(29)^.

No presente estudo, os pacientes não frágeis apresentaram maior mobilidade física (escore *Perme* de 27,6 pontos), seguidos dos pré-frágeis (escore *Perme* de 25,9) e frágeis (escore *Perme* de 15,2 pontos). No entanto, não foram encontrados estudos na literatura que permitissem discussões sobre essa relação entre o *Perme* e a condição de fragilidade. A literatura apresenta exclusivamente estudos referentes à avaliação da mobilidade física mediante o *Perme*, bem como aqueles que associaram o escore *Perme* às condições clínicas, como transplante hepático^(30)^, cirurgia cardíaca^(31)^, traqueostomia^(32)^ e intoxicação por organofosforados^(33)^.

A maioria dos estudos desenvolveu-se no ambiente da UTI, local com maiores restrições para caminhadas, e visou identificar o escore obtido pelos pacientes. Um exemplo é o estudo metodológico realizado com 50 pacientes de uma UTI coronariana na Turquia, apresentando média de idade 69 anos. Os resultados apontam para a média do escore *Perme* de 22 (5)^(34)^. Esse valor é próximo ao obtido neste estudo (22,9), o qual, no entanto, realizamos em enfermarias de hospital especializado no atendimento a pessoas idosas.

Outro estudo, desta vez conduzido fora do ambiente de UTI, usou uma coorte retrospectiva de 69 pacientes com covid-19 para avaliar a mudança de mobilidade durante a hospitalização. O escore médio do *Perme* na admissão à enfermaria foi de 17,5 (IC95% 15,8-19,3), aumentando para 24,8 (IC95% 23,3-26,3) na alta hospitalar. Verificou-se um incremento médio de 7,3 pontos (IC95% 5,7-8,8; p < 0,001) na mobilidade funcional dos pacientes durante o período de internação, demonstrando significativa melhora entre a admissão e a alta^(35)^. Quanto ao aumento da mobilidade funcional, destaca-se que eram pacientes com covid-19, o que envolve aspectos bastante distintos dos apresentados em outros motivos de internações.

Embora a maioria dos estudos concentre a aplicação do *Perme* na UTI, algumas pesquisas usaram-no em outros cenários^(30,35)^. Tal fato demonstra a possibilidade de aplicá-lo como uma ferramenta de avaliação da mobilidade em variados ambientes de cuidado em saúde e diversas condições clínicas, além de possibilitar a análise da evolução da mobilidade durante a hospitalização. A mobilização precoce deve ser sempre indicada, respeitando as contraindicações e limitações de cada paciente.

O comprometimento da mobilidade contribui para o processo de fragilização e potencializa eventos adversos durante a hospitalização^(18,19)^. Embora o distúrbio da mobilidade seja frequente, nem a disfunção da mobilidade nem a do equilíbrio são suficientes para definir a fragilidade. A mobilidade deve ser mensurada rotineiramente, já que muitas pessoas idosas perdem o condicionamento físico durante a hospitalização. Ainda, as avaliações regulares de mobilidade devem ser praticadas para monitorar a doença e a recuperação, especialmente nas pessoas idosas mais velhas. Essas questões tornam-se cada vez mais importantes em consequência do aumento da necessidade de hospitalização de idosos, sobretudo os frágeis.

Os resultados deste estudo mostram que pessoas idosas hospitalizadas classificadas como frágeis apresentam mobilidade significativamente reduzida; esse fato alinha-se com diretrizes internacionais, que apontam a perda de força e função muscular como elementos centrais da fragilidade^(17)^. Os achados reforçam a importância de identificar e intervir precocemente em pacientes pré-frágeis, pois estes ainda mantêm níveis de mobilidade semelhantes aos não frágeis, indicando uma janela de oportunidade para prevenção. Entretanto, o contexto hospitalar cria desafios adicionais, como barreiras à mobilização e limitações dos instrumentos de avaliação, sugerindo a necessidade de adaptações das recomendações internacionais para a prática clínica em enfermarias. Dessa forma, integrar a triagem sistemática a protocolos de mobilização adaptados pode melhorar o prognóstico funcional desses pacientes.

A utilização do *Perme* é importante como instrumento de avaliação da mobilidade em distintos contextos de atenção à saúde e diversas condições clínicas. Também se mostra como instrumento importante para analisar a evolução da mobilidade durante a hospitalização.

### Limitações do estudo

Destaca-se como limitação do estudo a lacuna encontrada na literatura sobre pesquisas que analisam a relação entre fragilidade e mobilidade física. Esse déficit restringiu as discussões dos resultados pertinentes a tais variáveis.

O viés de informação foi minimizado com estudo criterioso do método, além de treinamento dos entrevistadores, calibração dos instrumentos de coleta e apoio de especialista em estatística durante as fases de análise e interpretação dos dados. Foi ainda mitigado com o desenvolvimento de um protocolo de coleta, medição e interpretação de informações. Quanto à lacuna da literatura, procedeu-se a uma revisão de escopo sobre o instrumento de avaliação da mobilidade, o *Perme*.

### Contribuições para a Área

Os resultados deste estudo podem contribuir com a melhoria no gerenciamento do cuidado à pessoa idosa hospitalizada, uma vez que os dados ressaltam a importância de um cuidado fundamentado na avaliação e mobilização precoce durante a hospitalização. Tal gerenciamento torna se mais efetivo com o olhar de uma equipe multiprofissional, que deve estar atenta aos fatores associados à diminuição da mobilidade, entre os quais está a fragilidade física, frequentemente associada a desfechos negativos durante a hospitalização.

## CONCLUSÕES

Pessoas idosas frágeis hospitalizadas em enfermaria apresentaram menor mobilidade quando comparadas a indivíduos não frágeis ou pré-frágeis. Tal relação não foi observada na comparação entre os participantes não frágeis e pré-frágeis.

A menor mobilidade das pessoas idosas frágeis hospitalizadas é um alerta para a necessidade de desenvolver protocolos de mobilização precoce. Isso porque o déficit e/ou limitação da mobilidade contribui para a fragilidade física, que leva ao declínio funcional e às incapacidades nesse segmento etário.

## Data Availability

Os dados de pesquisa estão disponíveis em repositório: https://doi.org/10.48331/scielodata.MTY89M.
